# Bayesian modeling of the effect of vaccination and the delta and omicron variants on the COVID-19 epidemic in Burkina Faso using poisson log-linear autoregressive model

**DOI:** 10.3389/fpubh.2025.1622791

**Published:** 2025-09-11

**Authors:** Serge M. A. Somda, Issouf Traore, Bernard E. A. Dabone

**Affiliations:** ^1^Unité de Formation et de Recherche en Sciences Exactes et Appliquées, Université Nazi BONI, Bobo - Dioulasso, Burkina Faso; ^2^Centre de Méthodologie et de Gestion des Données, Centre MURAZ, Institut National de Santé Publique (INSP), Bobo - Dioulasso, Burkina Faso

**Keywords:** Bayesian Modeling, poisson model, log linear auto-regressive model, COVID-19 infection, COVID-19 variants, COVID-19 vaccination, Burkina Faso

## Abstract

**Introduction:**

Since its emergence in Burkina Faso in March 2020, the COVID-19 epidemic has undergone several shifts in its trajectory. These fluctuations have been influenced by government control measures, socio-economic dynamics, and biological mutations of the virus. However, the individual impacts of these factors remain insufficiently assessed, and the detailed history of the outbreak is not fully understood. This study aims to objectively evaluate the effects of two major viral variants (Delta and Omicron) as well as the introduction of vaccination on the epidemic's progression.

**Methods:**

We used publicly available surveillance data and adopted a Bayesian modeling framework, incorporating a Poisson counting process with log-linear autoregressive component. The events of interest were modeled as dummy time series to estimate their influence.

**Results:**

Our findings indicate that the emergence of the Delta variant was associated with an increase in weekly infections, while the arrival of the Omicron variant coincided with a decrease in case numbers. The vaccination was associated with an increase of transmission during the study period.

**Discussion:**

This work contributes valuable insights into the epidemic's dynamics in a West African context and offers important lessons for the design of future public health responses.

## 1 Introduction

The COVID-19 pandemic has been one of the most significant global health crises in recent history, altering the trajectory of public health systems, economies, and societies across the globe. The virus, first identified in December 2019 in Wuhan, China, quickly spread to multiple countries. On January 31, 2020, the World Health Organization (WHO) declared COVID-19 a Public Health Emergency of International Concern. A few weeks later, in March 2020, the outbreak was officially classified as a global pandemic (1).

Burkina Faso confirmed its first COVID-19 case in early March 2020. Shortly thereafter, the country experienced a rapid and exponential increase in the number of cases, just like what was seen in other parts of the world (2). This surge placed immense pressure on the already fragile healthcare infrastructure in Burkina Faso and raised urgent questions about preparedness and response strategies in West African nations.

As of July 4, 2022, the World Health Organization reported 550 million confirmed COVID-19 cases worldwide, with over 6 million deaths (3). In Africa, the pandemic led to nearly 12 million cases and around 254,000 deaths. Specifically in Burkina Faso, 21,044 cases were reported along with 387 deaths, reflecting a relatively smaller scale compared to global figures, but still representing a significant public health burden for the country.

Governments and public health authorities around the world implemented a variety of interventions to mitigate the spread of the virus and reduce mortality. These included lockdowns, mask mandates, testing and contact tracing protocols, as well as vaccination campaigns. However, in countries like Burkina Faso, the impact of these interventions was difficult to quantify due to complex and overlapping events, variability in public adherence, and challenges in data collection and management. As a result, accurately evaluating the effectiveness of specific interventions became a considerable challenge (4).

To address these complexities, a number of modeling studies were undertaken to better understand the dynamics of the COVID-19 epidemic in Burkina Faso. At the onset of the crisis, Guiro et al. (5) applied a classical Susceptible-Infected-Recovered (SIR) model to estimate key epidemiological parameters. This foundational approach was later extended to 16 West African countries by Honfo et al. (6), enabling a broader regional perspective. In parallel, statistical modeling approaches began to emerge. Konane and Traore (7) and Petropoulos et al. (8) developed time-series models to capture temporal patterns and make short-term forecasts. In the same time, Somda et al. (2) proposed a Bayesian framework to generate data-driven estimates, offering a probabilistic understanding of the epidemic's evolution.

With the WHO's declaration in May 2023 that COVID-19 was no longer a Public Health Emergency of International Concern, new questions emerged about the long-term effectiveness of various public health responses. In particular, there was increasing interest in developing locally tailored estimations of epidemiological indicators, rather than relying solely on generalized global models. Somda et al. (9) highlighted the importance of context-specific modeling, demonstrating that local (African) estimates could significantly improve the planning and implementation of health responses in African countries.

One of the most pressing post-crisis research questions concerns the interpretation of epidemic waves and their relationship with the emergence of viral variants. In Burkina Faso, two variants of concern were particularly impactful: the Delta variant (*B.1.617.2*) (10, 11), and the Omicron variant (*B.1.1.529*) (12, 13). These variants introduced new challenges by altering the shape and severity of the epidemic curves. The Delta variant was associated with increased transmissibility and higher mortality, while Omicron, though generally less severe, led to significant surges in number of case. These variant-driven waves often obscured the observable effects of public health interventions and thus became key confounders in evaluating policy effectiveness.

Another major event that influenced the trajectory of the pandemic was the introduction and widespread administration of COVID-19 vaccines. Several types of vaccines were approved for use in Burkina Faso and distributed across the population. Despite this, uncertainties remained about their real-world effectiveness in the local context. Vaccine hesitancy, logistical challenges, and the varying efficacy of different vaccine types all contributed to a complex and multifaceted public health landscape.

In this study, we aim to describe the effects of these significant events, particularly the emergence of the Delta and Omicron variants, and the roll out of vaccination on the COVID-19 epidemic in Burkina Faso. By leveraging a comprehensive dataset encompassing the entire course of the pandemic and applying a Bayesian statistical modeling approach, we seek to provide a nuanced understanding of how these factors influenced the spread of the virus. Our objective is to isolate and quantify the individual contributions of these events to changes in case numbers and mortality, thereby offering insights that could inform future epidemic response strategies both in Burkina Faso and across the broader West African region.

## 2 Methods

### 2.1 Data source

This study relied on publicly accessible data to analyze the progression of the COVID-19 epidemic in Burkina Faso and to estimate the impact of key events, namely the introduction of vaccination and the emergence of viral variants. The dataset was obtained from the World Health Organization (WHO) global COVID-19 database, available at: https://covid19.who.int/WHO-COVID-19-global-data.csv. This dataset provides a daily time series of epidemic indicators for all WHO member states, beginning in January 2020. Specifically, it includes the number of newly confirmed COVID-19 cases, cumulative confirmed cases, newly reported deaths, and cumulative deaths.

For the purpose of this study, we extracted data corresponding to Burkina Faso from the WHO dataset, and grouped them to epidemiological weeks. The number of new cases every week were the primary outcome variables. These indicators were selected because they represent the most direct measure of epidemic intensity and public health impact. They also allow for the evaluation of both disease transmission dynamics and mortality trends over time.

To assess the impact of major epidemic events on the evolution of the disease in Burkina Faso, we introduced binary (dummy) variables representing the occurrence of three key milestones: the introduction of vaccination, the appearance of the Delta variant, and the detection of the Omicron variant. Each dummy variable was set to 0 prior to the corresponding event and to 1 from the date of occurrence onward. This modeling approach allows us to estimate the discrete effects of each event while controlling for the underlying temporal structure of the epidemic.

The national COVID-19 vaccination campaign officially began on June 3, 2021. From that date forward, the vaccination dummy variable was assigned a value of 1. The Delta variant (*B.1.617.2*) was first identified in Burkina Faso on November 16, 2021. Accordingly, the Delta variant dummy variable takes the value 1 starting from this date. The Omicron variant (*B.1.1.529*) was subsequently confirmed in the country on January 7, 2022, and the corresponding dummy variable was activated from that date.

**Figure 1:**
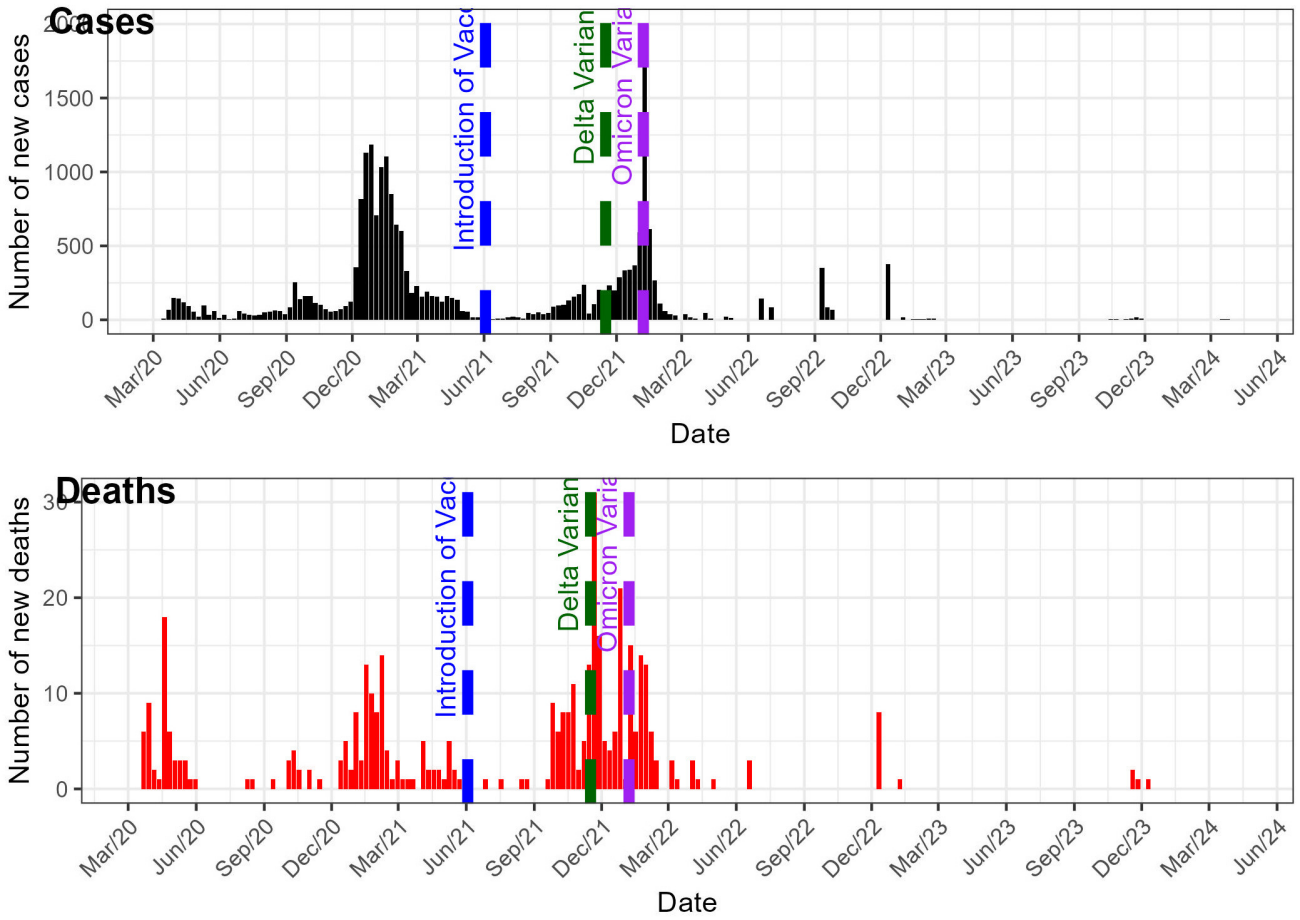
Evolution of the number of new COVID-19 cases and new COVID-19 deaths in Burkina Faso since 2020.

To provide a visual representation of the epidemic dynamics over time, we present the evolution of the number of new COVID-19 cases and deaths in Burkina Faso in Figure 1. This figure highlights the trends observed from the start of the pandemic through its later stages, offering insight into the major peaks and troughs that shaped the course of the outbreak.

### 2.2 Model description

To assess the effect of vaccination and the emergence of SARS-CoV-2 variants on the progression of the COVID-19 epidemic in Burkina Faso, we adopted a Bayesian statistical framework. Specifically, we modeled the daily number of new confirmed COVID-19 cases using a Poisson autoregressive process, allowing us to capture both temporal dependencies and the influence of external interventions or events.

Let *Y*_*t*_ represent the observed number of new COVID-19 cases on day *t*. We assume that *Y*_*t*_ follows a Poisson distribution with a time-varying rate parameter λ_*t*_, as shown in Equation 1:


(1) $$\begin{array}{l} Y_{t}\sim \text{Poisson}\left(\lambda_{t}\right) \end{array}$$

The Poisson distribution is a natural choice for count data and has been widely used in epidemic modeling due to its interpretability and analytical tractability. Building on this foundation, Fokianos and Tjøstheim (14) proposed a log-linear Poisson autoregressive model that offers greater flexibility than simple linear models.


(2) $$\begin{array}{l} \lambda =\alpha +\beta \log\left(1+Y_{t-1}\right)+\gamma \lambda_{t-1} \end{array}$$

In this specification, the parameter β captures the dependence on the previous number of new cases, providing short-term temporal dependence. The parameter γ represents the dependence on the expected growth rate, capturing long-term effects and allowing the model to exhibit long memory properties. The log-linear specification also permits negative dependence relationships (14). Given that infection counts typically follow exponential growth patterns driven by the basic reproduction number (9), Agosto and Giudici (15) proposed a modified version that was subsequently adopted by Kharroubi (16):


(3) $$\begin{array}{l} \log\left(\lambda_{t}\right)=\alpha +\beta \log\left(1+Y_{t-1}\right)+\gamma \log\left(\lambda_{t-1}\right) \end{array}$$

This formulation is particularly well-suited for modeling infectious disease time series, as it incorporates both short-term and long-term dependencies on past observations while accounting for potential intervention effects through interaction terms.

To estimate the effects of covariates on disease transmission dynamics, we extend the model by incorporating elasticity coefficients. This yields the following log-linear autoregressive equation:


(4) $$\begin{array}{r} \log\left(\lambda_{t}\right)=\alpha +\left(\beta +\nu_{1}\Sigma_{t}+\nu_{2}\Delta_{t}+\nu_{3}O_{t}\right)\log\left(1+Y_{t-1}\right)\\ +\left(\gamma +\mu_{1}\Sigma_{t}+\mu_{2}\Delta_{t}+\mu_{3}O_{t}\right)\log\left(\lambda_{t-1}\right) \end{array}$$

In this equation:

- α, β and γ are baseline parameters capturing the intercept, the dependence on previous observed cases (*Y*_*t*−1_) and the long-term autoregressive effect of past rates (λ_*t*−1_), respectively.
- Σ_*t*_, Δ_*t*_ and *O*_*t*_ are binary indicator variables, denoting the presence of the vaccination campaign, the Delta variant, and the Omicron variant, respectively. Each takes the value 0 before the event and 1 afterward.
- ν_1_, ν_2_, ν_3_, are interaction coefficients that quantify how the vaccination and the two variants modulate the short-term (case-based) dependence structure.
- μ_1_, μ_2_, μ_3_, are coefficients capturing how these events affect the long-term (rate-based) dependence in the epidemic process.

This modeling framework provides a flexible approach to capture how COVID-19 transmission dynamics evolve as a function of both internal epidemic momentum and critical external interventions. The elasticity coefficients enable direct interpretation of covariate effects on transmission patterns. For example, a positive value of ν_2_ (the coefficient for the Delta variant covariate) would indicate that the emergence of Delta intensified short-term transmission dynamics by increasing the dependence on previous case counts. Conversely, a negative value of μ_1_ (associated with vaccination coverage) would suggest that vaccination campaigns effectively reduced long-term epidemic growth by dampening the persistence of the transmission rate over time.

To complete the Bayesian specification, we assigned weakly informative priors to all model parameters. Specifically, we assumed independent normal priors centered at zero with large variance, as show below:


(5) $$\begin{array}{l} \alpha ,\beta ,\gamma ,\nu_{i},\mu_{i}\sim N\left(0,10^{6}\right)\;i=1,2,3 \end{array}$$

These non-informative priors reflect our intent to let the data primarily drive the posterior distributions of the parameters, avoiding strong assumptions about their likely values. This is particularly important in settings like Burkina Faso, where limited prior information about the epidemic's progression and the real-world effects of interventions is available.

### 2.3 Simulation and material

Parameter estimation for the Bayesian model was conducted using a Markov Chain Monte Carlo (MCMC) simulation approach. Given the complexity of the model and the need for flexibility in specifying hierarchical structures and prior distributions, we employed the BUGS (Bayesian inference Using Gibbs Sampling) framework, implemented via the JAGS (Just Another Gibbs Sampler) engine (17, 18). The analysis was conducted using the R statistical programming language, which offers seamless integration with JAGS through packages such as *rjags* and *coda*.

The MCMC simulation was run for a total of 12,000 iterations. To ensure convergence and eliminate the influence of initial values, the first 2,000 iterations were discarded as burn-in. The remaining 10,000 iterations were retained for posterior inference. Trace plots and autocorrelation diagnostics were inspected to assess convergence and mixing of the chains. Thinning was applied to reduce autocorrelation in the posterior samples.

## 3 Results

### 3.1 Incidence of COVID-19

The primary outcomes assessed were the daily number of incident cases.

**Figure 2:**
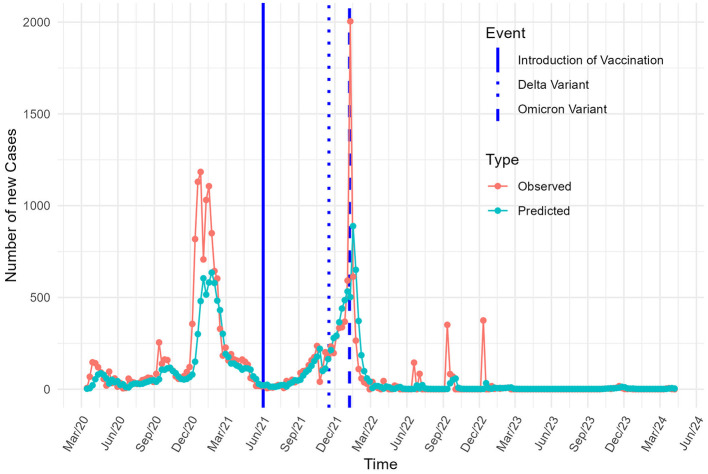
Observed and predicted COVID-19 cases in Burkina Faso.

Figure 2 presents the goodness-of-fit diagnostics, comparing observed and predicted values for COVID-19 cases. The model successfully captures the overall temporal trends in incident cases, although it tends to underestimate the amplitude of peak transmission periods. Nevertheless, the predicted trajectory aligns well with the observed pattern, indicating a satisfactory fit for modeling new infections.

Turning to the parameter estimates, Table 1 summarizes the median and the 95% credibility interval of the posterior distributions for each key model component. These include baseline (intercept) terms, autoregressive effects, and the short- and long-term influences of vaccination, Delta, and Omicron on incident cases.

**Table 1:** Posterior estimates of key model parameters for COVID-19 incidence (Median, 95% CI).

**Parameter**	**Short term**	**Long term**
Overall intercept	0.099 (0.093, 0.105)	
Specific intercept	0.505 (0.501, 0.509)	0.442 (0.437, 0.447)
Vaccination	0.015 (0.010, 0.020)	0.031 (0.026, 0.036)
Delta variant	0.007 (0.002, 0.012)	0.022 (0.017, 0.027)
Omicron variant	−0.035 (−0.040, −0.029)	−0.022 (−0.026, −0.017)

### 3.2 Effect of the different major events during the course of the epidemic

**Figure 3:**
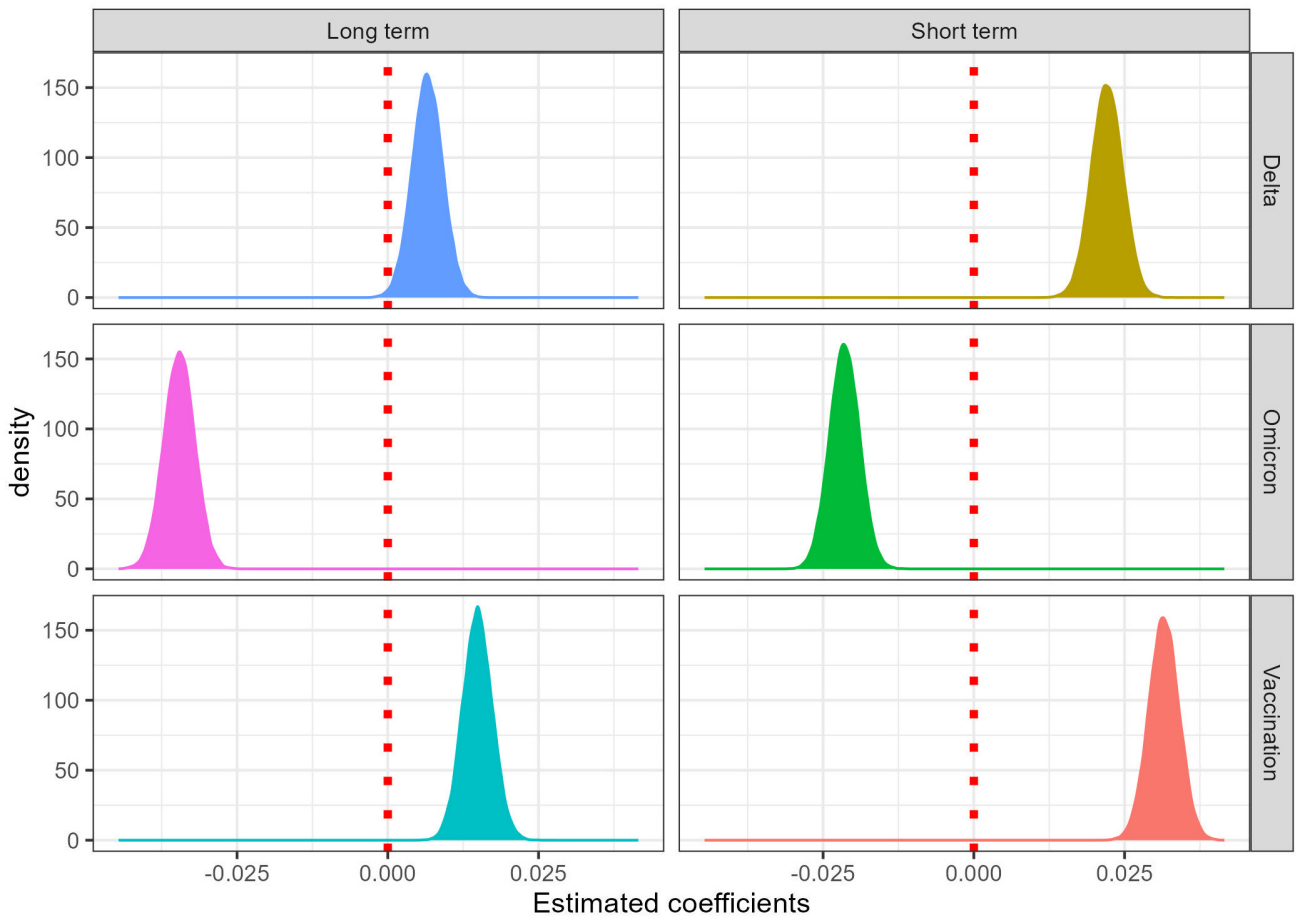
Distribution of the model parameters related to COVID-19 incidence.

The influence of each of the three key events (vaccination, and the emergence of the Delta and Omicron variants) on the COVID-19 epidemic curve was assessed by examining the posterior distributions of the corresponding parameters. These are visualized in Figure 3, which presents the estimated short- and long-term effects of each event on the number of new COVID-19 cases in Burkina Faso.

The first major intervention analyzed was the introduction of the COVID-19 vaccination campaign. Contrary to expectations, both the short-term and long-term effects of vaccination on incidence appear to be significantly positive. This suggests that, rather than being immediately associated with a reduction in the number of new cases, the vaccination period coincided with a rise in case numbers.

The emergence of the Delta variant (*B.1.617.2*) in Burkina Faso, marks the second major event in the epidemic timeline. The model reveals that the short-term and long-term effects of Delta on incidence were both positive, indicating an increase in the number of reported cases following its appearance.

The third event analyzed was the detection of the Omicron variant (*B.1.1.529*) in Burkina Faso. The model results indicate a negative effect on incidence, with both short-term and long-term parameters significantly less than zero.

## 4 Discussion and conclusion

The model developed in this study provides a framework for understanding the association of key epidemic events, namely, the introduction of COVID-19 vaccination and the emergence of two major SARS-CoV-2 variants (Delta and Omicron), with the evolution of incident cases in Burkina Faso. Through a Bayesian autoregressive approach, we estimated the short- and long-term effects of these events, allowing us to evaluate their real-world impact on the epidemic trajectory in this West African context.

### 4.1 Interpretation of vaccination effects

The introduction of COVID-19 vaccines was globally anticipated as a turning point in pandemic control. In Africa, however, vaccine roll out occurred under challenging circumstances: limited access, logistical barriers, public skepticism, and competing public health priorities. Our analysis surprisingly identified a statistically significant positive association between vaccination and the number of new cases. This finding contrasts with expectations and raises important questions about the contextual and behavioral dynamics surrounding vaccine introduction in Burkina Faso.

Previous mathematical modeling studies have attempted to assess the projected impact of vaccination in Africa. For instance, Montcho et al. (19) used a deterministic compartmental model across eight African countries and concluded that achieving 60% vaccination coverage would reduce the effective reproduction number below one. Similarly, Mathebula et al. (20) found that increased vaccination was associated with reduced cumulative deaths. However, these approaches relied on strong assumptions and lacked empirical data validation. To our knowledge, no previous data-driven statistical analysis has evaluated the effect of vaccination in Africa using real epidemic time series.

The seemingly counterintuitive association between vaccination and increased COVID-19 cases in Burkina Faso likely reflects the complex socio-political context surrounding vaccine rollout. Several interconnected factors may explain this finding. First, the official vaccination campaign commenced on June 3, 2021, coinciding with the relaxation of several public health restrictions including curfews, school closures, and bans on mass gatherings. This temporal overlap confounds the direct assessment of vaccination effects. Second, public optimism regarding vaccination's protective effects likely fostered risk compensation behavior, where individuals increased social activity and reduced adherence to other preventive measures. This behavioral shift could have facilitated viral transmission even as vaccination rates remained low. Third, the WHO reports that by August 2024, only 27.1% of Burkina Faso's population had received at least one dose and 23.2% were fully vaccinated–levels far below those necessary to achieve population-level protection. Without adequate coverage, vaccines cannot effectively interrupt transmission chains.

This interpretation aligns with prior studies. Moghadas et al. (21) estimated that vaccination reduced transmission by only 4.6%, with greater effectiveness in preventing severe disease than infection. Similarly, Wu et al. (22) and Troiano and Nardi (23) emphasized that vaccines are more efficient at reducing hospitalizations and deaths than in stopping infection, and that public doubts about vaccine effectiveness may have contributed to both low uptake and inconsistent results.

The consistency between short- and long-term vaccination effect distributions suggests this phenomenon persisted over time without notable temporal variation, supporting the interpretation that structural factors rather than temporary effects drove the observed association.

### 4.2 Variant effects and epidemic dynamics

The COVID-19 epidemic in Africa has been influenced by multiple factors beyond basic transmission dynamics (4). Several studies have documented epidemic waves following the emergence of new viral variants (24), with the Delta and Omicron variants playing particularly significant roles in shaping transmission patterns.

Our results show that the Delta variant was associated with increased new cases, particularly through elevated growth rates. These findings align with epidemiological evidence that Delta demonstrated higher transmissibility and virulence than earlier strains. Despite ongoing public health measures and increasing population immunity, Delta likely contributed to renewed infection waves through its enhanced transmission characteristics.

Surprisingly, Omicron emergence was associated with decreased new cases in our model. This finding appears inconsistent with global observations of Omicron's exceptional transmissibility. Several factors may reconcile this apparent contradiction. First, while Omicron demonstrated increased transmissibility, it was generally associated with milder disease severity, potentially resulting in reduced healthcare-seeking behavior and case detection. Second, by Omicron's emergence, vaccination coverage had expanded and substantial population immunity from previous waves had accumulated, potentially moderating its apparent epidemiological impact. Finally, the model structure may not fully disentangle overlapping effects of vaccination scale-up and variant emergence, potentially attributing some vaccine-related benefits to the Omicron period rather than to immunization efforts.

### 4.3 Modeling deaths

The model was also run on mortality, over the study period. Unfortunately, this model did not reach to stable results. Our modeled events did not exert a measurable impact on the mortality curve in Burkina Faso. There are several plausible explanations for the model's inability to detect a pattern in COVID-19 mortality. First, the absolute number of deaths in Burkina Faso remained relatively low throughout the pandemic. With 387 deaths officially reported by July 2022, the resulting data may have been too sparse to support robust statistical inference. In Bayesian terms, the likelihood function would provide limited information to update the prior distributions, especially for events occurring later in the epidemic timeline.

Second, mortality data in low-resource settings can be subject to underreporting and misclassification. Many COVID-19 deaths may have occurred outside health facilities or been attributed to other causes due to limited testing or diagnostic capacity, especially in rural areas. Conversely, misattributions or rumors of COVID-19 deaths have also circulated, creating doubts on the figures published by the health authorities. All this further reduce the signal-to-noise ratio in the mortality series, weakening the model's ability to detect real associations.

Third, it is also possible that the events under consideration did not substantially affect mortality. For example, the Omicron variant, although highly transmissible, is generally associated with milder clinical outcomes compared to Delta. Similarly, while vaccination campaigns may not have reduced case numbers in the short term, they could have helped prevent severe disease and death, effects that may not be adequately captured due to limitations in data granularity and lag structures.

In light of these findings, we opted not to interpret the mortality model results at all. The absence of statistically significant effects does not necessarily imply a true lack of impact; rather, it may reflect the limitations of the data and the complex interplay of factors that influence COVID-19 mortality. Further investigation using more detailed clinical and demographic data such as age, comorbidities, and vaccine type could help clarify these relationships and improve the model's sensitivity.

Beyond the current approach, exploring alternative modeling strategies such as zero-inflated Poisson or log-normal models could prove beneficial for deaths modeling. The efficacy of these models would be enhanced by incorporating time-dependent covariates, including data on non-pharmaceutical measures, hospitalization trends, and vaccine coverage. Nevertheless, achieving this would require extensive data collection and curation, along with a focused analysis at a finer geographic scale, specifically at the region or district level.

### 4.4 Challenges and limitations

While our model offers a novel statistical approach to epidemic analysis in a low-resource setting, several limitations must be acknowledged.

First, data quality is a critical concern. Like many countries in the region, Burkina Faso faced challenges in accurate case detection and mortality reporting. Limited access to testing, underdiagnosis, and social stigma led to potential underreporting of both cases and deaths. Moreover, reporting backlogs led to data irregularities, necessitating the aggregation of daily observations into weekly counts. Second, the potential for confounding is substantial. As discussed earlier, multiple factors such as movement restrictions, public space closures, and natural immunity may have influenced transmission independently of the studied events. Additionally, while climate and seasonality were not explicitly modeled due to a lack of evidence in Burkina Faso, they remain plausible sources of confounding. Third, there is significant variability in coverage both in terms of vaccine uptake and the spread of different variants which may have influenced the observed effects. Ultimately, the model specification may be subject to criticism. The decision to treat changes as dummy variables without including interaction terms limits the ability to account for the duration of vaccination or the emergence of new variants. Different lag structures can be also used but we did not find any reference in the litterature. Alternative approaches, such as interrupted time series analyses or different posterior distribution specifications, could be considered. Some alternative scenarios were tested but did not yield significantly different results. Further investigations are planned. These may include models that account for mortality outcomes, alongside those already examined, and that integrate additional covariates or control factors to better isolate causal effects.

## Data Availability

The original contributions presented in the study are included in the article/supplementary material, further inquiries can be directed to the corresponding author.
